# Delayed Hemorrhage Followed by Vertebral Artery Injury during Cervical Anterior Controllable Antedisplacement and Fusion Surgery: Case Report and Literature Review

**DOI:** 10.1111/os.13413

**Published:** 2022-08-05

**Authors:** Tong Yongjun, Xie Yaming, Chen Biao, Yang Yonghong, Zhao Xinhua

**Affiliations:** ^1^ Department of Orthopaedics Zhejiang Hospital Hangzhou China

**Keywords:** Angiography, Anterior controllable ante‐displacement and fusion, Hemorrhage, Ossification of the posterior longitudinal ligament, Vertebral artery injury

## Abstract

**Background:**

Vertebral artery injury (VAI) during cervical spine surgery is rare. Anterior controllable ante‐displacement and fusion (ACAF) surgery is a novel technique for treating degenerative cervical spine disorders, especially ossification of the posterior longitudinal ligament. To date, there have been no reports of VAI during cervical ACAF surgery. Here, we report a rare case of perioperative complication of VAI during ACAF surgery. The available English literature that provides treatment instructions were reviewed.

**Case Presentation:**

A patient diagnosed with mixed ossification of the posterior longitudinal ligament (OPLL) underwent ACAF surgery from C2–C6. Two level transverse foramina were ruptured, and severe bleeding was encountered during ACAF osteotomy. Hemostatic tamponade was performed using bone waxes. The patient had delayed hemorrhage on postoperative day 6. Emergence angiography revealed two vertebral artery pseudoaneurysms in the ruptured transverse foramina. A balloon‐expandable covered stent was deployed to treat the pseudoaneurysm. The patient recovered without complications.

**Conclusion:**

ACAF surgery is a good choice for multiple‐level OPLL disease, but special attention should be paid to VAI in the perioperative period. Intraoperative tamponade with bone wax and postoperative digital subtraction angiography (DSA) are effective in preventing disaster‐related hemorrhage.

## Graphical abstract

we present a rare case of VAI during cervical anterior controllable antedisplacement and fusion (ACAF) surgery from C2–C6, which was complicated by a postoperative vertebral pseudoaneurysm with delayed hemorrhage. Intraoperative VAI was temporarily controlled using bone wax tamponade. The patient experienced severe artery bleeding after a cough due to pseudoaneurysms which was resolved by deploying a stent inside the VA. ACAF surgery is a good choice for multiple‐level OPLL disease, but special attention should be paid to VAI in the perioperative period. Intraoperative tamponade with bone wax and postoperative DSA are effective in preventing disaster‐related hemorrhage.

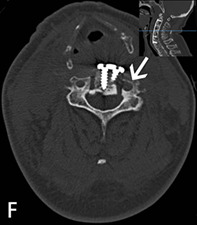

## Introduction

Ossification of the posterior longitudinal ligament (OPLL) of the cervical spine is a severe disease which is characterized by one or multiple‐segment ligament ossification, and the incidence reported in the literature is between 2%–4% in Asian populations and 0.01%–2% in non‐Asian populations.^1^ Varying degrees of neurologic symptoms can be present including radiculopathy and myelopathy in cervical OPLL patients.^2^

Treatment of cervical spondylopathy caused by OPLL is a major challenge. There is still much controversy regarding the choice of surgical approach in these patients.^1^ Vertebral corpectomy via the anterior approach decompresses the spinal nerve directly by removing the ossified ligament which shows satisfactory outcomes with a high risk of cerebrospinal fluid leakage.^3^, ^4^ Laminoplasty or laminectomy via the posterior approach can provide adequate indirect decompression of the spinal cord by expanding the spinal canal and has low risk of dural tear. However, the decompression effect may gradually diminish due to the progression of OPLL.^5^ Axial pain remains a major problem after the posterior approach.^3^, ^4^

The anterior controllable antedisplacement and fusion (ACAF)^6^ of cervical spine technique (or vertebral body sliding ostectomy,VBSO^7^) is a relatively new technique for the treatment of multi‐segment or upper cervical ossification of the posterior longitudinal ligament. The technique was developed in recent years, and its outcomes are relatively satisfactory.^8^

The vertebral artery is vital for the brain's blood supply. The vertebral artery is at potential risk of injury during cervical spine surgery due to its anatomic location which runs near the surgical site.^9^ Vertebral artery injury (VAI) during cervical spine surgery is a rare but serious complication,^10^ with studies reporting an incidence ranging from 0.07%^11^–0.14%,^12^ and most VAIs encountered during cervical spine decompression or instrumentation.^11^ However, there have been no reports of VAI during cervical ACAF surgery. Here we present a rare case of VAI during cervical ACAF surgery, which was complicated by a postoperative vertebral pseudoaneurysm with delayed hemorrhage. Intraoperative VAI was temporarily controlled using bone wax tamponade. The patient experienced severe artery bleeding after a cough due to pseudoaneurysms which was resolved by deploying a stent inside the VA.

## Case Report

### Clinical Presentation and Examination

A 55‐year‐old woman presented with complaints of severe neck pain, and leg coordination problems without numbness and radiating pain, which the patient experienced for 2 years. The patient complained of gradually developing a feeling of heaviness and weakness in both legs along with an inability to walk at a brisk pace during the last 2 years. The patient further reported the development of persistent, electric shock‐like shooting pain, with numbing that began at the shoulders and radiated down both arms to the 1st and 2nd digits three months after an accidental head injury.

On examination, the cervical spine was found to be stiff. The pain was localized at the cervical paraspinal muscles. The cervical spurling test was positive for bilateral reproduction of upper‐extremity pain. The sensations in the patient's bilateral upper forearms and in their 1st and 2nd digits were decreased. The patient had a strength score of 5/5 in their C5–C6 distribution, and Hoffmann's sign in both hands was positive. The patient's knee and ankle reflexes were hyper‐reflexive and their Babinski reflexes were present bilaterally.

**Fig. 1:**
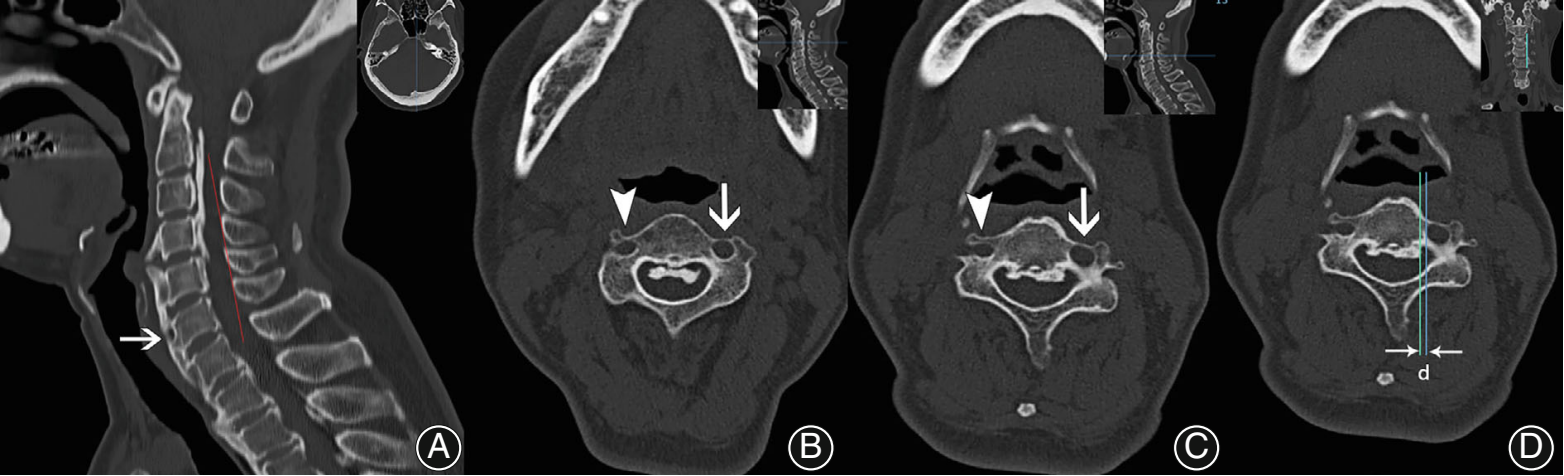
Cervical CT scan demonstrates a mixed OPLL from C2–C6. (A) Sagittal reconstruction CT. (B, C) Axial CT cuts of C3 and C4. The K‐line is positive (A, red line), with evident anterior multi‐segmental calcification (A, arrow) that is indicative of DISH. The ossific ligament, occupying 1/3 of the cervical spine canal in some segments, has a broad base attached to the posterior edge of the vertebral bodies (B, C). The transverse CT scans show the morphological difference between the left and right transverse foramina at C3 (B, right, arrowhead; left, arrow) and C4 (C, right, arrowhead; left, arrow: medial erosion of the vertebrae due to artery tortuosity can be observed). The left transverse foramina of C4 is significantly bigger and more closed to the ipsilateral uncinate process tip than the right side (D, the blue line indicates the border of the internal cortex of transverse foramina of C4 in the transverse cut; the green line indicates the ipsilateral uncinate process tip; the distance (d) between the two lines is 1.1 mm). DISH, diffuse idiopathic skeletal hyperostosis

**Fig. 2:**
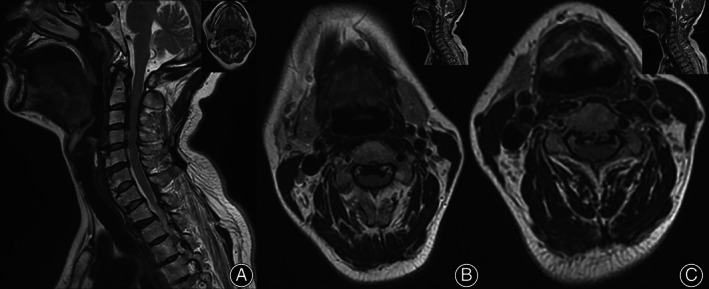
(A) Sagittal cervical MRI T2 view demonstrates multi‐level stenosis from C2–C6. (B–C) The axial view shows the spinal canal being occupied more than 1/3 in some segments by the ossific ligaments, along with lateral recess stenosis

Computed tomography (CT) of the cervical spine demonstrated prominent extrusion ossification of the posterior longitudinal ligament (OPLL) extending from C2 to C6 (Figure. 1A–C). The ossific ligaments occupied nearly 1/3 of the cervical spinal canal in some segments. In addition, continuous anterior calcification of several cervical and thoracic vertebral bodies was observed in the sagittal reconstruction slices, indicating diffuse idiopathic skeletal hyperostosis (DISH)(Figure 1A). The left transverse foramina of C4 is significantly bigger and more closed to the ipsilateral uncinate process tip than the right side (Figure 1D). The patient's cervical MRI also revealed multiple levels of cervical spinal stenosis (Figure 2).

### Surgical Procedure

The patient underwent cervical spinal ACAF. The ACAF process has been reported previously.^13^ After general anesthesia and neurophysiologic monitoring were performed, the patient was in a supine position with the neck fully extended. A right Smith–Robinson incision was made to expose the anterior cervical spine. After confirming the surgical level using radiological plain radiography, discectomies were performed from C2 to C6. The proper volumes of the posterior portion of the C2 level and the anterior vertebral bodies of C3–C5 were resected with a high‐speed drill to facilitate hoisting of the vertebrae‐OPLL complex. Subsequently, bilateral osteotomies of C3–C5 were performed.

### Intraoperative Results

**Fig. 3:**
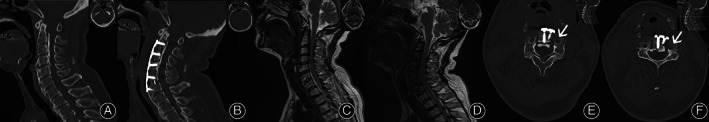
Cervical CT and MRI on postoperative day 1. Compared to the preoperative images, the cervical spine canal is obviously enlarged due to elevation of the vertebrae‐OPLL complex (A *vs* B; C *vs* D). Axial slices at C3 (E, arrow) and C4 (F, arrow) show that the medial cortices of the transverse foramina are violated at the V2 segments of the VA

Owing to the widened base of the OPLL, the troughs of the left C3–C4 vertebrae were created beyond the uncinate process tip, and significant bleeding was encountered indicating violation of VA at the V2 segment (Figure 3). Hemostatic tamponade was performed immediately with digital pressure and with bone wax. After the bleeding was controlled, intervertebral cages filled with autogenic bone were placed at C2–C6 levels. An appropriate‐length pre‐curved cervical plate was then fixed at the C2 and C6 vertebrae for temporary fixation. Finally, the screws were gradually tightened in each vertebral body at the same pace to achieve anterior hoisting of the vertebrae‐OPLL complex. The fascia and skin were sutured layer by layer.

**Fig. 4:**
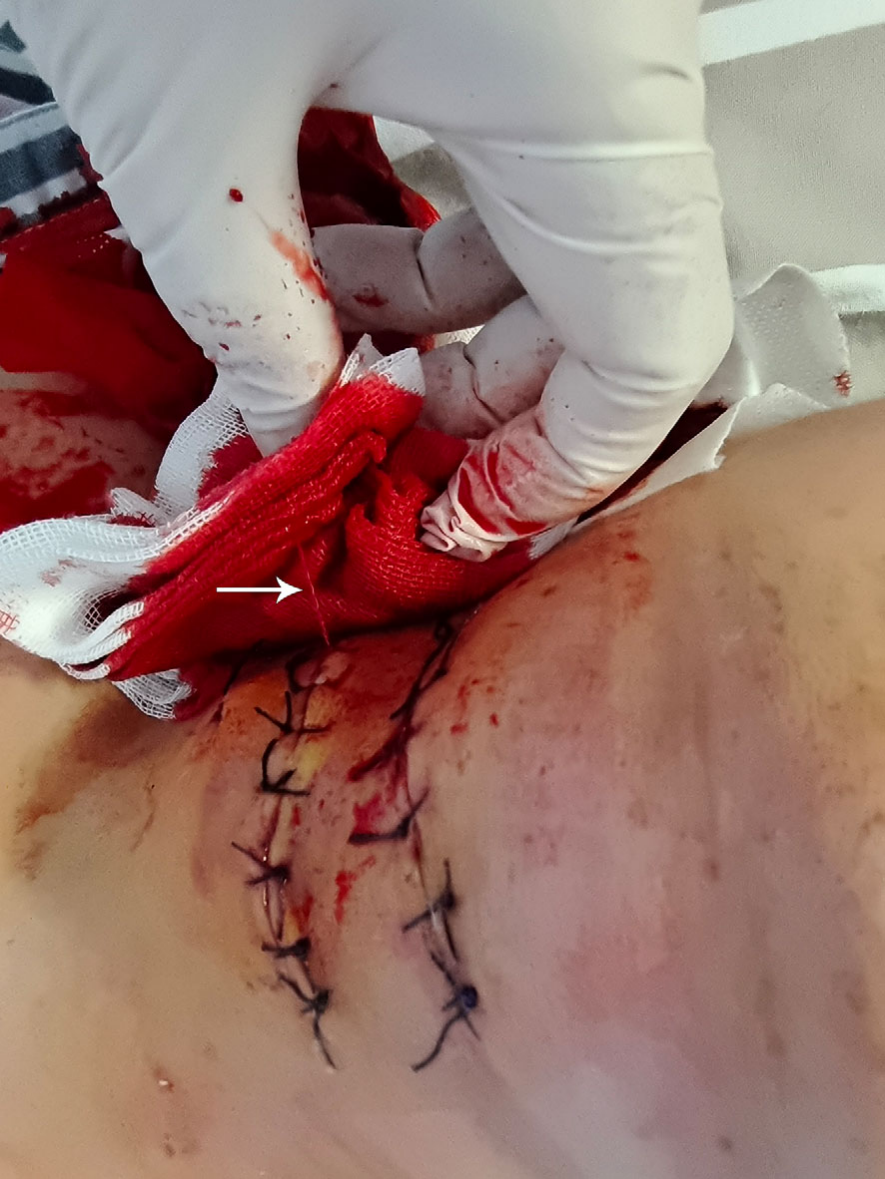
Image showing the patient's cervical swelling, with a red spurting stream of blood (arrow) coming out of their neck incision on postoperative day 6.

On postoperative day 6, the patient experienced sudden swelling of their neck incision after coughing. Meanwhile, a spurting stream of blood was detected from the neck incision (Figure 4). The patient's blood pressure and SPO_2_ continuously decreased as a result of hematoma compression. After emergency tracheotomy and endotracheal intubation were performed, the patient was transferred to an operating room for an endovascular assessment.

**Fig. 5:**
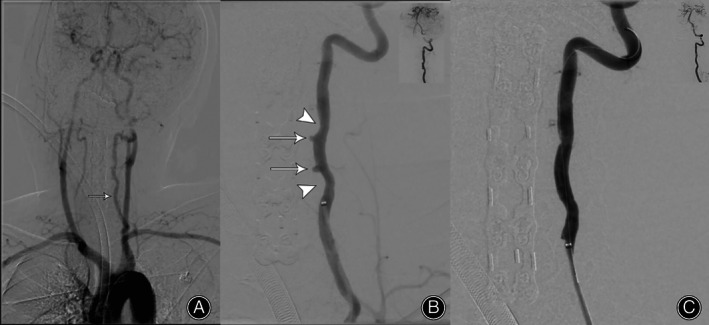
Intraoperative angiography showing that the left vertebral artery was dominant (A, arrow); two vertebral artery pseudoaneurysms (B, arrow) are evident in the left ruptured transverse foramina at C3 and C4 with no contrast extravasation. There are two incurved notches (B, arrowhead) next to the two pseudoaneurysms, indicating that the ruptured medial transverse foramina wall is filled with bone wax. The intraoperative fluoroscopy radiograph shows that after the stent was fully deployed, the two pseudoaneurysms were completely resolved, with no significant stenosis and good flow in the left VA (C)

An angiogram of the vertebral artery showed that the left side was dominant (Figure 5A). Two vertebral artery pseudoaneurysms were detected in the left ruptured transverse foramina at C3 and C4 (Figure 5B). The decision was made to use a stent to seal the two pseudoaneurysms and keep the left VA patent. Under fluoroscopic guidance, a balloon‐expandable covered stent was placed across the laceration site and deployed; the two pseudoaneurysms were completely covered and later disappeared (Figure 5C).

### Postoperative results

**Fig. 6:**
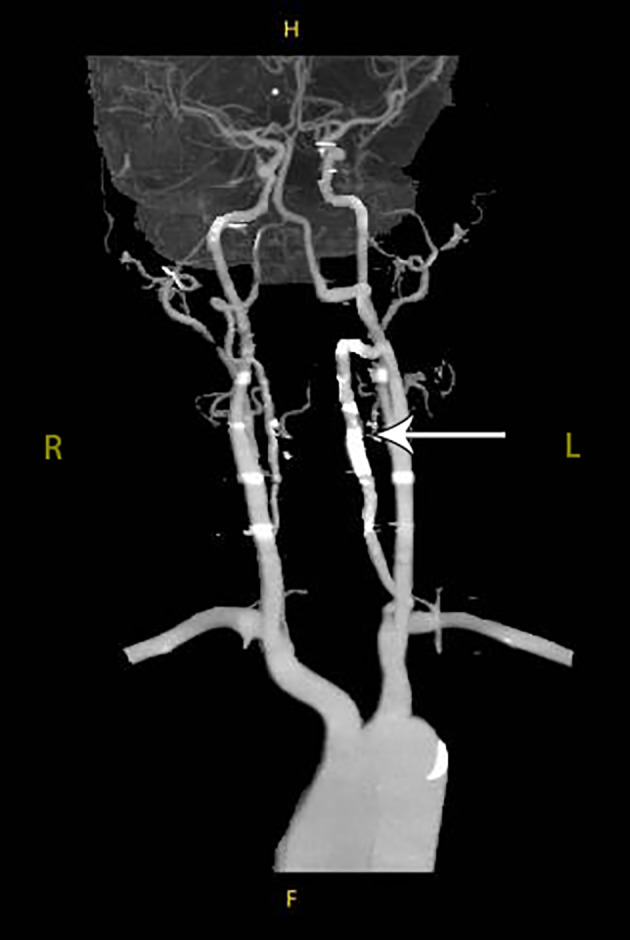
Three‐month follow‐up CTA of the vertebral artery showing a similar diameter of the lumen of the left vertebral artery with good flow compared to the preoperative status (arrow), with no residual pseudoaneurysm formation. CTA, CT angiography

The patient recovered after the angiography without any complications. The patient's neurological symptoms were effectively improved at the 3‐month follow‐up. CT angiography (CTA) of the vertebral artery revealed a similar diameter of the lumen of the left vertebral artery with good flow compared to the preoperative status (Figure 6).

## Discussion

The VAs, two vital vessels of the brain, are the first branch of the subclavian arteries. The VA can be separated into four segments^14^: the V1 segment travels between the longus scolli and anterior scalene muscles and then courses anterior to the transverse foramen of C7; the V2 segment, also known as foraminal segment, typically begins when the VA enters the transverse foramen of C6 and ends at C2. The V1 and V2 segments are the most vulnerable during anterior cervical spine surgery.^14^

VA injury can cause devastating neurologic impairment or catastrophic bleeding. Lee reported that severe neurologic sequelae developed in as many as 23% of VAIs.^15^ Most vertebral artery injuries are iatrogenic and often encountered during anterior or posterior cervical surgery; the remaining VAI cases are secondary to blunt traumatic injuries such as cervical spine dislocation.^12^

ACAF is a novel surgical technique that has been introduced in recent years. This technique is widely applied to treat cervical ossification of the posterior longitudinal ligament (OPLL).^16^ To our knowledge, this is the first reported case of vertebral artery injury occurring during ACAF, which resulted in the delayed hemorrhage of a vertebral pseudoaneurysm.

### Reasons for ACAF Surgery

This patient had multi‐segment cervical spinal stenosis with reasonable cervical alignment and a positive K‐line, which made them a good candidate for the posterior approach based on previous literature.^17^, ^18^ However, we performed ACAF surgery in this patient for the following reasons: (i) The base of the ossified ligament was wide, and part of it approached the intervertebral foramen; aposterior approach decompression of spinal canal was indirect for cervical stenosis, which would make it inadequate for neural nerve compression relief; (ii) Posterior laminoplasty or laminectomy was mostly performed at C3–C7 segments.^19^ However, for the ossific ligament originating from the odontoid process of C2 in this patient, C2 dome‐like expansive laminoplasty was insufficient for decompression, while extending C1–C2 laminoplasty had potential complications including loss of cervical curvature and increased cervical anteversion; (iii) Reports of axial pain^4^ and C5 palsy^20^ following the posterior approach are more common than the anterior approach. Studies have reported that ACAF can preserve better cervical alignment, expand the spinal canal area more efficiently, improve Japanese Orthopaedic Association (JOA) score, reduce perioperative complications compared to the tradition posterior approach.^20^, ^21^, ^22^

### Methods of Preventing Intraoperative Vertebral Artery Injury

Kong *et al*.^23^ found in a cadaveric study that in normal vertebrae, the uncinate process tip is located medial to the transverse foramen. The distance between the uncinate process tip and the medial wall of the ipsilateral transverse foramen (UP tip‐TF distance) was the smallest at C4 (3.16 ± 0.54) and increased cranially and caudally. Osteotomy slotting performed within the tip of the uncinate process has a low risk of VAI. However, Curylo *et al*. reported in a cadaveric study that the incidence of tortuous vertebral artery course was 2.7%, with the transverse foramina located 0.14 mm medial to the Luschka joint.^24^ The preoperative CT scan of this case showed that both C3 and C4 had wide ossific ligament bases (Figure 1B,C), while the left transverse foramen was very close to the ipsilateral uncinate process tip (Figure 1D). A wide‐based OPLL and tiny UP tip‐TF distance left the VA at high risk during lateral osteotomies when using the uncinate process tip as a landmark.

According to our experiences and to literature reviews, for patients with a wide base and tiny UP tip‐TF distance, the following suggestions are recommended:

- All patients who are candidates for ACAF should undergo radiological assessments to determine the morphology of the transverse foramina via cervical CT reconstruction before surgery; this is to evaluate any variation within the transverse foramina and determine the UP tip‐TF distance and width of the base of the ossific ligament.
- Slotting should be performed vertically first, followed by wedging. Kong *et al*. suggested that for wide‐base cases, a slot should be created vertically to the posterior vertebral wall and a 1‐mm Kerrison rongeur should be used to remove the ossification laterally until the margin of the OPLL effectively reduces the VA risk.^23^
- Osteotomy with Piezosurgery. The content of the transverse foramina is soft and significantly different from that of the surrounding hard bone. A normal drill cuts the bone and soft tissue indiscriminately using high‐speed rotation. Low frequency Piezosurgery enables the cutting of mineralized structures, not soft tissue. Piezosurgery stops cutting when the slotting violates the transverse foramina wall and soft tissue is encountered, enabling the early detection of transverse foramina violations and avoiding vertebral artery injury.
- Intraoperative radiology should be performed before osteotomy. One recent study reported that ACAF surgery had a steep learning curve; specific errors were encountered in 20.9% of cases in the early phase (within 29 cases), including but not limited to oblique osteotomy and insufficient isolation of the vertebrae‐OPLL complex.^25^ Although there were no VA injuries following ACAF, according to the high error rate reported, we strongly recommend that surgeons who have less experience in ACAF perform intraoperative radiology before lateral osteotomy to make sure that the slotting is not beyond the tip of the UP, especially in patients with tortuous course VAs.

### Recommended Treatment Strategy for Vertebral Artery Injury According to Literature Reviews

Treatment of VA injuries is complicated. Depending on whether it is dominant, iatrogenic VAI has many treatment options, such as tamponade, endovascular intervention, direct repair, or ligation^12^, ^14^, ^26^, ^27^, ^28^(Table 1). Several studies have reported strategies for treating intraoperative VAI.^9^, ^12^, ^14^, ^29^ Hemostatic tamponade is an initial procedure involving digital pressure, bone wax, gelfoam, or cottonoid.^27^, ^28^, ^30^ Subsequently, bleeding assessments should be performed.

**TABLE 1:** Treatment of iatrogenic vertebral artery injury during anterior cervical spine surgery reported in the literature

Authors	Size	Diagnosis	Surgical procedure	Treatment	Complication
Burke^10^	6	Cervical spondylopathy (6)	ACDF (2), Corpectomy (4)	Tamponade (3), repair (2), ligation (1)	Intraoperative death (1), PICA infarct (1)
Lunardini^11^	53	NA	ACDF (10), corpectomy (26), anterior release (1), anterior exposure (8), anterior foraminotomy (4), anterior instrumentation (4)	Tamponade (7.4%), repair (13.2%), ligation (29.4%), embolization (11.7%), stenting (5.9%), NA (32.3%)	Temporary neurologic sequelae (3), cerebellar infarct (6), death (5), NA (1)
Neo^12^	5	Cervical spondylopathy (3), cervical spondylopathy +OPLL (1), NA (1)	ACDF (4), anterior foraminotomy (1)	Tamponade (4), embolization (1)	Screw loosening (1), pharyngeal discomfort (1), hoarseness (1), NA (1)
Smith^26^	10	Cervical spondylopathy (4), tumor (2), OPLL (1), nonunion of fracture (1), osteomyelitis (1)	Corpectomy (7), hemi‐corpectomy (2), discectomy (1)	Tamponade (3), repair (3), ligation (4)	Vertigo (1), PICA/CSF fistula repaired (2), muscle weakness (3), all resolved at final following‐up
Maughan^27^	7	cervical spondylopathy (4), vertebral fracture (2), odontoid fracture (1)	ACDF (4), corpectomy (2), odontoid screw (1)	Tamponade (1), repair (2), ligation (1), embolization (3)	Hemiparesis and dysmetria (1), resolved at 42nd month
Golfinos^28^	4	Cervical spondylopathy (4)	NA	Repair (3), ligation (1)	No complication (4)
Hsu^30^	7	NA	NA	Tamponade (1), repair (1), ligation (1), embolization (1), stenting (2), NA (1)	NA

Abbreviations: ACDF, anterior cervical decompression and fusion; CSF, cerebrospinal fluid; NA, not available; OPLL, ossification of the posterior longitudinal ligament; PICA, posterior inferior cerebellar artery.

If the bleeding is controlled, and an open repair is feasible, the primary repair should be completed.^29^ If an open repair is not feasible, an intraoperative endovascular team should be consulted, if available. For dominant VA, repair/stenting should be performed; otherwise, embolization could be a feasible method for bleeding control. If an intraoperative endovascular intervention team is not available, but bleeding is controlled with tamponade, an immediate postoperative endovascular assessment should be performed to exclude delayed hemorrhage and pseudoaneurysm formation.^12^

If the bleeding is not controlled, an intraoperative endovascular intervention team should be consulted if available. The patient could be treated with either vessel reconstruction/stent or embolization, depending on the dominance of the injured VA. If an intraoperative endovascular intervention team is not available, the VA should be ligated by exposing the proximal and distal sides.

Ligation is a safe method to halt bleeding in most VA injury cases^9^, ^12^, ^30^; however, ligation is associated with significant morbidity and mortality when a dominant VA injury is encountered.^12^, ^31^ Preoperative VA assessment is important to determine the dominant VA, and every surgical candidate should undergo this examination.

### Specific Treatments of This Case

For the patient discussed here, angiographic CTA was not performed to evaluate the dominant vertebral artery. However, the preoperative axial CT showed that the diameter of the transverse foramina on the left side was significantly larger than that on the right side (Figure 1B,C). We speculated that the left side was the dominant artery, which was confirmed using postoperative digital subtraction angiography (DSA, Figure 5A). Therefore, we did not attempt to ligate the ruptured artery. Instead, hemostatic tamponade was performed immediately using digital pressure and tamponade with bone wax. Fortunately, the bleeding was halted by the bone wax tamponade.

Our experience with VAI during anterior cervical surgery is that when a vertebral artery injury is encountered in the V2 segment, tamponade with bone wax can effectively stop the bleeding intraoperatively, after which angiography can further be performed to reconstruct or occlude the VA depending on whether it is the dominant side or not. As we can see from the postoperative angiography, there are two indented notches next to the two pseudoaneurysms, indicating ruptured medial transverse foramina wall filled with bone wax (Figure 5B).

This patient experienced delayed hemorrhage after a severe cough on postoperative day 6. Angiography detected two vertebral artery pseudoaneurysms in the left ruptured transverse foramina at C3 and C4 (Figures 3E,F and 5B). We speculate that temporary bone wax filling within the rupture of transverse foramina wall could fall off over time due to deficient osteogenic ability. After the bone wax partially falls off, VAs with high pressure are prone to protruding from the local defect of the transverse foramina wall, where a pseudoaneurysm subsequently forms (Figure 5B). The pseudoaneurysm ruptures when its pressure increases suddenly, such as following a severe cough, which finally causes delayed bleeding (Figure 4). Many studies have reported delayed hemorrhage^32^ and pseudoaneurysms^33^ after temporary tamponade of VAI, which is consistent with this case. A systematic literature review found that the incidence of pseudoaneurysms after temporary tamponade with VAI can be as high as 48%.^9^ Therefore, immediate postoperative vertebral artery angiography or CTA is mandatory after iatrogenic VAI to reduce the incidence of devastating consequences, even if the bleeding has been well‐controlled by tamponade intraoperatively. We evaluated the location and size of the vertebral aneurysm using angiography and determined whether it needed to be treated to prevent possible delayed bleeding. Although there was no contrast extravasation during angiography, we deployed one stent at the laceration site of the VA to prevent further hemorrhage.

The patient's neurological symptoms effectively improved at the 3‐month follow‐up. Postoperative CTA showed a similar diameter of the lumen of the left vertebral artery with good flow compared to the preoperative status and no residual pseudoaneurysm formation (Figure 6).

### Conclusion

Vertebral artery injury during ACAF is a relatively rare but catastrophic complication. In patients who undergo ACAF surgery, special attention should be paid to the UP tip‐TF distance and any abnormities of transverse foramina. ACAF surgery should be carefully scheduled according to the dominance of the VA, abnormities of transverse foramen, the UP tip‐TF distance, and the width of the base of ossific ligament. If there is obvious VAI during surgery, temporary tamponade with bone wax could efficiently halt the hemorrhage. Vertebral angiography after surgery must be performed as soon as possible to detect the presence of a pseudoaneurysm and deploy stents, if necessary.

## Authors' Contributions

Tong Yongjun: Conceptualization, Methodology, Writing—Original draft preparation, Writing— Reviewing and Editing. Xie Yamin.: Data curation, Writing—Reviewing and Editing, Software. Chen Biao: Software, Visualization. Yang Yonghong: Resources, Visualization, Investigation. Zhao Xinhua: Conceptualization, Supervision. Tong Yongjun^†^ and Xie Yamin^†^ should be considered joint first author.

## Declaration of Interest Funding and Acknowledgments

The authors declare that there is no conflict of interest. The manuscript submitted does not contain information about medical device(s)/drug(s). No funds are received in support of this work.
